# Porcine Hemagglutinating Encephalomyelitis Virus Enters Neuro-2a Cells via Clathrin-Mediated Endocytosis in a Rab5-, Cholesterol-, and pH-Dependent Manner

**DOI:** 10.1128/JVI.01083-17

**Published:** 2017-11-14

**Authors:** Zi Li, Kui Zhao, Yungang Lan, Xiaoling Lv, Shiyu Hu, Jiyu Guan, Huijun Lu, Jing Zhang, Junchao Shi, Yawen Yang, Deguang Song, Feng Gao, Wenqi He

**Affiliations:** a Key Laboratory of Zoonosis Research, Ministry of Education, College of Veterinary Medicine, Jilin University, Changchun, China; b Key Laboratory of Zoonosis Research, Ministry of Education, Institute of Zoonosis, Jilin University, Changchun, China; University of Texas Southwestern Medical Center

**Keywords:** porcine hemagglutinating encephalomyelitis virus, Rab, clathrin, endocytosis, neurovirulent coronavirus

## Abstract

Porcine hemagglutinating encephalomyelitis virus (PHEV) is a highly neurovirulent coronavirus that invades the central nervous system (CNS) in piglets. Although important progress has been made toward understanding the biology of PHEV, many aspects of its life cycle remain obscure. Here we dissected the molecular mechanism underlying cellular entry and intracellular trafficking of PHEV in mouse neuroblastoma (Neuro-2a) cells. We first performed a thin-section transmission electron microscopy (TEM) assay to characterize the kinetics of PHEV, and we found that viral entry and transfer occur via membranous coating-mediated endo- and exocytosis. To verify the roles of distinct endocytic pathways, systematic approaches were used, including pharmacological inhibition, RNA interference, confocal microscopy analysis, use of fluorescently labeled virus particles, and overexpression of a dominant negative (DN) mutant. Quantification of infected cells showed that PHEV enters cells by clathrin-mediated endocytosis (CME) and that low pH, dynamin, cholesterol, and Eps15 are indispensably involved in this process. Intriguingly, PHEV invasion leads to rapid actin rearrangement, suggesting that the intactness and dynamics of the actin cytoskeleton are positively correlated with viral endocytosis. We next investigated the trafficking of internalized PHEV and found that Rab5- and Rab7-dependent pathways are required for the initiation of a productive infection. Furthermore, a GTPase activation assay suggested that endogenous Rab5 is activated by PHEV and is crucial for viral progression. Our findings demonstrate that PHEV hijacks the CME and endosomal system of the host to enter and traffic within neural cells, providing new insights into PHEV pathogenesis and guidance for antiviral drug design.

**IMPORTANCE** Porcine hemagglutinating encephalomyelitis virus (PHEV), a nonsegmented, positive-sense, single-stranded RNA coronavirus, invades the central nervous system (CNS) and causes neurological dysfunction. Neural cells are its targets for viral progression. However, the detailed mechanism underlying PHEV entry and trafficking remains unknown. PHEV is the etiological agent of porcine hemagglutinating encephalomyelitis, which is an acute and highly contagious disease that causes numerous deaths in suckling piglets and enormous economic losses in China. Understanding the viral entry pathway will not only advance our knowledge of PHEV infection and pathogenesis but also open new approaches to the development of novel therapeutic strategies. Therefore, we employed systematic approaches to dissect the internalization and intracellular trafficking mechanism of PHEV in Neuro-2a cells. This is the first report to describe the process of PHEV entry into nerve cells via clathrin-mediated endocytosis in a dynamin-, cholesterol-, and pH-dependent manner that requires Rab5 and Rab7.

## INTRODUCTION

Porcine hemagglutinating encephalomyelitis virus (PHEV) is a nonsegmented, positive-sense, single-stranded RNA coronavirus that belongs to the genus Betacoronavirus, together with mouse hepatitis virus (MHV), human coronavirus OC43 (HCoV-OC43), severe acute respiratory syndrome coronavirus (SARS-CoV), and others (https://www.ncbi.nlm.nih.gov/Taxonomy/Browser/wwwtax.cgi?id=694002). PHEV was initially isolated from encephalomyelitic suckling piglet brains in 1962, and since then, several outbreaks of PHEV infection have been reported (1–4). Affected piglets exhibit vomiting and wasting disease (VWD) and/or encephalomyelitis, and the overall mortality rates for affected piglets under 3 weeks of age range from 30% to 100%. PHEV propagates via neural circuits to the central nervous system (CNS) and targets nerve cells as a site of replication, resulting in severe nerve damage (5, 6). Although major progress has been made in the past decades toward understanding the viral characteristics, many pathological aspects of this virus have yet to be characterized.

Coronaviruses (CoVs) are health threats to a wide variety of mammalian and avian species. The coronavirus spike (S) glycoprotein contains two distinctive domains, both of which can function as receptor-binding domains (RBDs) and recognize protein or sugar receptors (7). Therefore, the S protein is a major determinant of viral host range and is involved in viral binding and fusion, a set of processes that result in coronavirus entry (8). As for the coronaviral RBD located on the PHEV S protein, it was found to interact with the neural cell adhesion molecule (NCAM) and to participate in viral infection, suggesting that PHEV entry occurs via a receptor recognition pattern (9). Subsequent electron microscopic analysis demonstrated that membranous coating-mediated endo- and exocytosis can be employed by PHEV for its transsynaptic transfer (10). The evidence regarding the mechanism of PHEV entry has been very limited until now, and a detailed and comprehensive study of the endocytic mechanism involved in PHEV uptake was urgently needed.

Viral entry is the initial step toward a successful infection. Enveloped viruses achieve internalization via two primary pathways: some viruses appear to fuse directly with the plasma membrane, whereas others take advantage of the endocytic mechanism of the host (11). Most animal viruses gain access to target cells by hijacking host endocytic pathways, such as clathrin-mediated endocytosis (CME), macropinocytosis, caveola/raft-mediated endocytosis, clathrin- and caveolin/raft-independent endocytosis, and variations of these themes that are not yet well characterized (12, 13). CME, the most evolutionarily conserved endocytic pathway, is characterized by the uptake of cargo, such as the transferrin-iron complex, toxins, and viruses, by invaginations or clathrin-coated vesicles (CCVs) (14). It is the primary route for internalization of many viruses, including African swine fever virus (ASFV), vesicular stomatitis virus (VSV), and rabies virus (RABV) (15–18). During this process, low pH plays an essential role, and the dynamics of the endosomal system are tightly regulated by the small Ras related in brain (Rab) GTPases (19, 20). Another well-characterized pathway is caveola/raft-dependent endocytosis, where caveolae are defined as specialized lipid raft domains composed of caveolin and high levels of cholesterol (21). The pathway requires unique structural and signaling machinery and is involved in internalization of some envelope viruses, such as human immunodeficiency virus (HIV), human coronavirus 229E (HCoV-229E), and infectious spleen and kidney necrosis virus (ISKNV), among others (22–24). In addition, research in recent decades has found that some viruses can induce macropinocytosis as a direct endocytic route for productive entry and infection (25–28).

So far, how PHEV enters host cells to start a productive infection has not yet been explored. In the present work, we addressed the roles of different endocytic molecules and pathways involved in PHEV entry and intracellular trafficking in host cells. Thus, a combination of pharmacological and molecular techniques was carried out to allow the visualization, tracking, and localization of PHEV. The results indicate that CME is part of the PHEV infection process in host cells and that dynamin-2, Eps15, membrane cholesterol, low pH, and a dynamic actin cytoskeleton are specifically required. Additionally, activation of Rab5 significantly affected the early stages of PHEV infection and disturbed virus transportation in endocytosed vesicles at the postentry stages. Together, our data not only contribute greatly to the pathological characterization of the PHEV entry and intracellular trafficking route but also provide new insights into antiviral drug design.

## RESULTS

### Kinetics of PHEV invasion in Neuro-2a cells.

**FIG 1:**
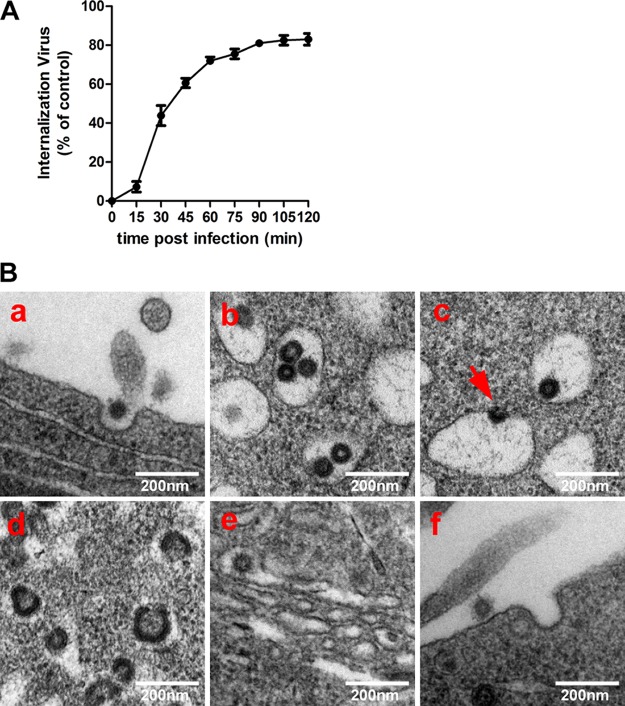
PHEV entry into Neuro-2a cells occurs via the endocytic pathway. (A) Entry kinetics of PHEV. Neuro-2a cells were incubated with PHEV, and noninternalized virus particles were inactivated with citrate buffer at the indicated time points. Infectivity was calculated by an IFA using an anti-PHEV-S antibody. (B) Ultrastructural analysis of PHEV entry and assembly in Neuro-2a cells. When cells were incubated with purified PHEV virions on ice, attached virus particles were engulfed by electron-dense membrane invaginations on the cell surface (a) and internalized into large endocytic vesicles, either individually or in groups (b and c). Subsequently, they were transported along the flow of endosomal maturation for uncoating, replication, and structure synthesis (d). Progeny virions were enclosed individually within small decorated vesicles in the ER-Golgi intermediate compartments (e) and then released from the cell through an opening at the fusion site (f).

The kinetics of PHEV infection was assessed by an immunofluorescence assay (IFA) using an anti-PHEV-S antibody in Neuro-2a cells. As the infectivity assay showed, the percentage of PHEV entry into Neuro-2a cells initially increased, rapidly reached the half-maximal level (50%), by 30 min after infection, and plateaued after 1 h (Fig. 1A). Based on these data, the time required for PHEV entry was estimated to be approximately 60 min under the conditions of the assay, and this value was used to interpret all PHEV internalization assays reported in this paper. To detect the course of PHEV entry visually, Neuro-2a cells were inoculated with purified PHEV virions for 30 min on ice, and we then employed thin-section transmission electron microscopy (TEM). We observed the attachment of virus particles along the cell surface before the cells were shifted to 37°C to allow entry; subsequently, PHEVs were engulfed by coated pits, which may represent primary endocytic vesicles (Fig. 1B, panel a). Endocytosed virions were mainly trapped within large vesicles in the cytoplasm, either individually or in groups (Fig. 1B, panel b), and the virus-containing coated vesicles were transported intracellularly, during which the virus fused with the endosome membrane and the nucleocapsid was released (Fig. 1B, panel c). Subsequently, viral replication and protein structure synthesis were widely observed at 90 min postinfection (Fig. 1B, panel d). The earlier sign of viral assembly observed inside the cytoplasm was that large numbers of virions with undecorated membranes were present in the dilated cisternae of the Golgi network (Fig. 1B, panel e). As the infection progressed, small coated vesicles, each tightly enclosing a single enveloped virion, were apparently fused with the plasma membrane. Progeny virions seemed to be released from the cell through an opening at the fusion site, but the membranous coating remained at the release sites (Fig. 1B, panel f). These findings raised evidence that membranous coating-mediated endo- and exocytosis can be used by PHEV for invasion and cellular transfer.

### PHEV enters endosomal structures and requires a low pH.

**FIG 2:**
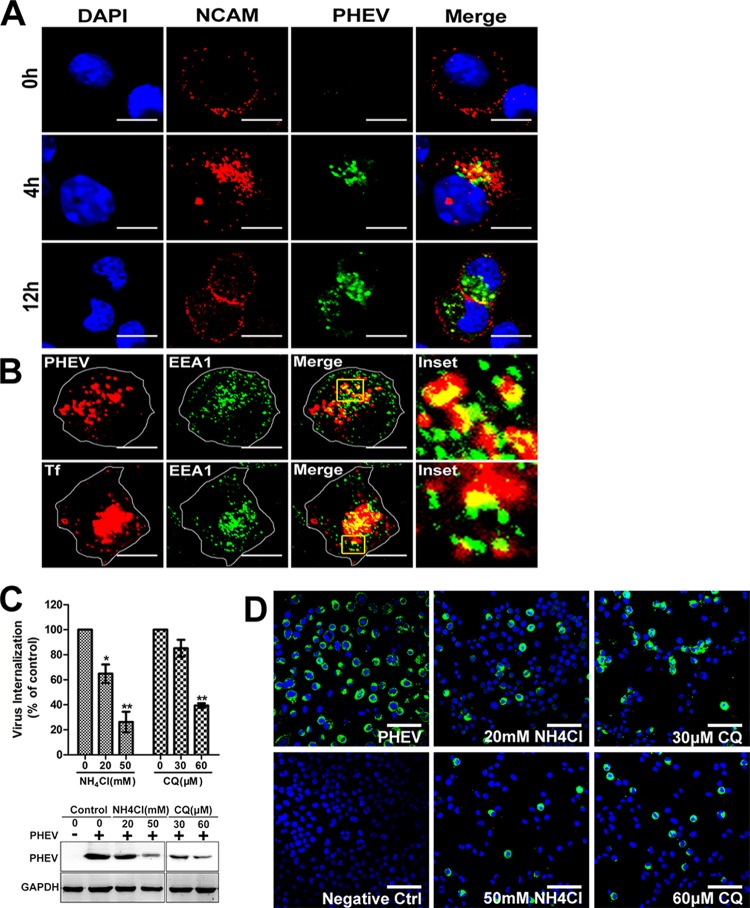
Inhibition of endosomal acidification prevents PHEV entry. (A) The PHEV receptor NCAM translocates with virus particles from the plasma membrane to cytoplasmic compartments. Neuro-2a cells were treated with PHEV for the indicated times at 37°C, and immunostaining was conducted using anti-NCAM and anti-PHEV antibodies. (B) PHEV cargos were internalized into EEA1-positive endosomes. Cells infected with PHEV were fixed and immunostained with anti-EEA1 and anti-PHEV antibodies. As a positive control, cells were probed with Tf-AF594 for 30 min at 37°C and then subjected to IFA. The insets show colocalization of PHEV or Tf with EEA1 inside the cytoplasm. (C) Neuro-2a cells were infected with PHEV in the presence of NH_4_Cl or CQ at the indicated doses for 1 h, after which the endocytosed viral loads were estimated by qRT-PCR and Western blotting. (D) Neuro-2a cells grown on coverslips were either left untreated or treated with NH_4_Cl or CQ for 1 h and subsequently incubated with PHEV. At 12 hpi, cells were fixed and stained with anti-PHEV-S primary antibodies. Bars, 10 μm (A and B) and 50 μm (D). *, *P* < 0.05; **, *P* < 0.01.

As previously reported, NCAM is a potential cellular receptor that interacts with the PHEV S protein, and this association is critical to PHEV infection (29). Neuro-2a cells were incubated with PHEV and fixed at the indicated time for an IFA using anti-NCAM and anti-PHEV-S antibodies. A dual staining assay for NCAM and PHEV showed that NCAM was localized primarily at the cell surface under normal conditions, but its expression was induced and the protein was transported from the plasma membrane to intracellular compartments after incubation with PHEV at 37°C for 4 h, whereas colocalization of these two proteins in the vesicles was observed in the perinuclear area (Fig. 2A). After an additional 8 h of incubation, NCAM-containing vesicles were less visible within the cytoplasm and instead were seen clustered near the cell surface, suggesting that the receptors were recycled, most likely by endocytosis (Fig. 2A). Early endosomal autoantigen 1 (EEA1) is a membrane tethering factor required for the fusion and maturation of EEs in endocytosis. To further confirm that PHEV enters Neuro-2a cells via endocytic pathways, dual immunostaining for PHEV and the early endosome (EE) marker protein EEA1 was performed. We observed a punctate pattern characterized by the indicated colocalization, suggesting that PHEV was internalized to EEs (Fig. 2B). As a positive control, we monitored trafficking of Alexa-labeled transferrin (Tf), which binds to its receptor and is internalized in coated vesicles, followed by fusion with endosomes (30). Once internalized within the primary endocytic vesicles, many viruses require acidic pH in the endosome to undergo uncoating and/or escape from the endosome, and the local pH drops following internalization into endolysosomes. To test whether PHEV entry is pH dependent, Neuro-2a cells were pretreated with the weakly basic amines NH_4_Cl and chloroquine (CQ), which selectively raise the luminal pH of the endosome and lysosome, and the cells were then infected with PHEV. In the presence of either 50 mM NH_4_Cl or 60 μM CQ, PHEV internalization decreased to less than 40% of the level found in untreated cells, while both reagents inhibited viral protein synthesis even at low concentrations (Fig. 2C). Moreover, the relative infection rates of PHEV as monitored by IFA were inhibited in a dose-dependent manner (Fig. 2D). These results reflect the normal traffic of membrane flow, indicating that internalized PHEV endocytic vesicles fuse with EEs and that the process occurs in a low-pH-dependent manner.

### Dynamin-2 dependence of PHEV internalization and infection.

**FIG 3:**
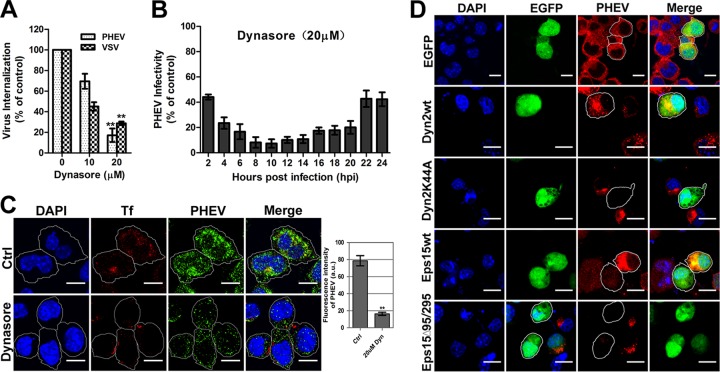
PHEV infection occurs in a dynamin-dependent manner. (A) Neuro-2a cells were pretreated with dynasore for 1 h at the indicated concentrations before PHEV infection, and the amount of virus endocytosed was measured by qRT-PCR at 2 hpi. (B) Neuro-2a cells were pretreated with 20 μM dynasore for 1 h, infected with PHEV for various times, and processed for qRT-PCR analysis. (C) PHEV-infected cells were pretreated with dynasore and given a pulse of Tf-AF488 for 30 min. Cells were fixed, and the uptake of transferrin and viral particles was quantified with a laser scanning confocal microscope. The mean fluorescence intensity (arbitrary units [a.u.]) of PHEV staining is represented in the histogram on the right. Bars, 10 μm. (D) Neuro-2a cells were transfected with EGFP or with EGFP-tagged wild-type dynamin-2 (Dyn2wt), DN mutant dynamin-2 (Dyn2K44A), wild-type Eps15 (Eps15wt), or DN mutant Eps15 (EpsΔ95/295). Twenty-four hours after transfection, cells were infected and processed for confocal microscopy analysis. The percentage of infected cells out of the number of successfully transfected cells was determined for each experiment. Three independent experiments were performed, and 30 infected and transfected cells per condition were scored for quantification analysis in each experiment. Bars, 20 μm. *, *P* < 0.05; **, *P* < 0.01.

Dynamin-2 (DNM-2), a 100-kDa GTPase that is responsible for endocytosis, plays an essential role in cellular membrane fission during vesicle formation and therefore is required for clathrin- and caveola-mediated endocytosis. Here we used dynasore, a cell-permeating noncompetitive inhibitor of dynamin, to ascertain whether PHEV infection is dynamin dependent. Neuro-2a cells were pretreated with 0, 10, or 20 μM dynasore for 1 h at 37°C before PHEV infection, and then the lysates were harvested for quantitative reverse transcription-PCR (qRT-PCR) assay. As a control, we monitored the impact of the inhibitor on infection with VSV, whose internalization was previously proved to occur in a clathrin- and dynamin-dependent manner. Treatment of Neuro-2a cells with 20 μM dynasore resulted in a decrease of over 80% in PHEV internalization (Fig. 3A), and the suppression lasted for over 24 h postinfection (Fig. 3B), implying that dynamin-2 might play a crucial role in multiple stages of the viral life cycle. When we further tested specialized functions with dynasore, treatment of cells with the chemical inhibitor blocked the uptake of fluorescently labeled transferrin, a cargo that is internalized via the dynamin- and clathrin-dependent endocytic mechanism (Fig. 3C). In this situation, we also observed a significant inhibition of the viral load in the cytoplasm of dynasore-treated cells relative to that for the control cells (Fig. 3C). We next explored the effect of the dominant negative (DN) K44A mutant of dynamin-2 (Dyn2K44A), which was previously described to have decreased GTPase activity resulting in reduced endocytosis (31). When Neuro-2a cells overexpressing EGFP-Dyn2K44A were infected 24 h later with PHEV, they showed a decrease of nearly 85% in PHEV infection (Fig. 3D). However, in control cells expressing either enhanced green fluorescent protein (EGFP) or wild-type dynamin-2 (Dyn2wt), normal infection was observed, as indicated by punctate staining in the cytoplasmic region. These findings indicate that PHEV endocytosis is dependent on dynamin-2 functionality.

### Clathrin-mediated endocytosis is involved in PHEV entry.

**FIG 4:**
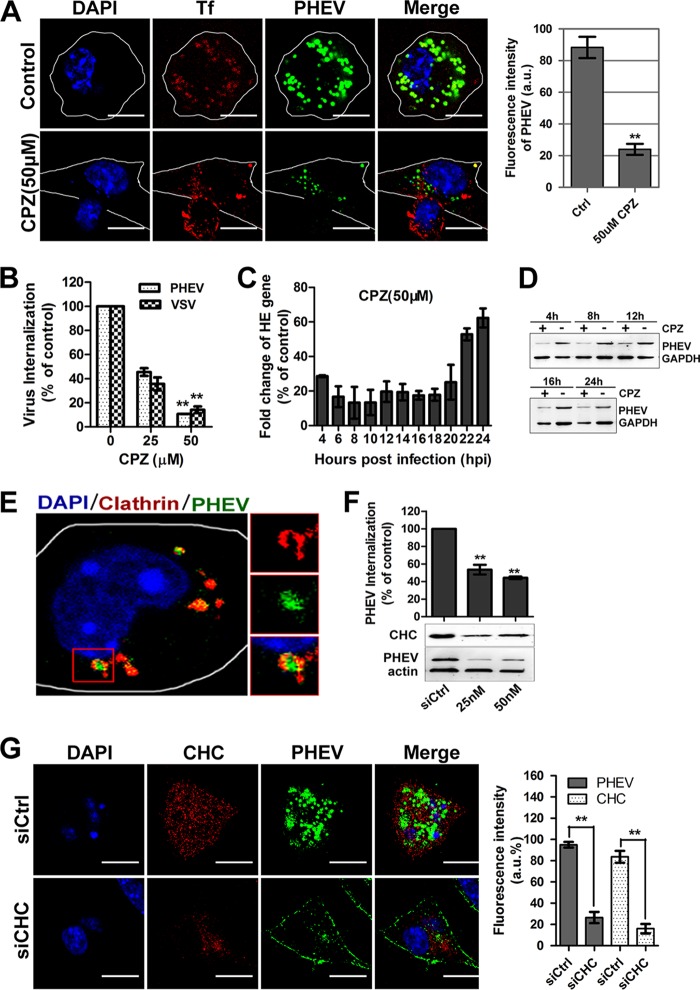
PHEV infection occurs via a CME-dependent mechanism. (A) The infectivity of PHEV was assessed in Neuro-2a cells that were either mock treated or treated with CPZ (50 μM). The infected cells were fixed and visualized by confocal microscopy. (B) A PHEV internalization assay was carried out with cells pretreated with CPZ at the indicated concentrations. Following internalization, surface-bound viruses were removed and detected by qRT-PCR. The internalization of VSV via CME was used as a control. (C) PHEV RNA replication was inhibited by pretreatment of the cells with CPZ for 1 h, and relative hemagglutinin-esterase (HE) gene expression was assessed using qRT-PCR. (D) PHEV protein synthesis was suppressed by pretreatment with CPZ. Cell lysates were collected, and the expression level of the major viral S protein was measured by Western blotting. (E) PHEV particles colocalized with clathrin. Neuro-2a cells were transfected with DsRed-clathrin prior to incubation with PHEV. The enlarged box indicates PHEV particles (green) that were swallowed into the vesicle structure, which bears clathrin (red) on the outer surface; colocalized signals were widely observed using confocal microscopy. (F) Neuro-2a cells were transfected with an siRNA directed against CHC or a control mock siRNA (siCtrl). Inhibition of PHEV internalization and replication was assessed by qRT-PCR to quantify relative gene expression (top) or by immunoblotting to analyze viral protein synthesis (bottom). The RNAi efficiency against CHC was also examined using an anti-CHC primary antibody. (G) The PHEV load and CHC expression in Neuro-2a cells against a background of CHC depletion were calculated at 24 hpi by confocal microscopy. The relative fluorescence intensities of PHEV and CHC staining are presented in the histogram on the right. Bars, 10 μm. **, *P* < 0.01.

To better understand the pathway used by PHEV to enter Neuro-2a cells, we compared its sensitivities to inhibitors of different endocytic mechanisms. Chlorpromazine (CPZ) is a specific inhibitor commonly used to block the CME pathway by preventing the assembly of clathrin-coated pits (CCPs) at the plasma membrane and causing clathrin lattices to assemble on endosomal membranes (32). Cells treated with CPZ were incubated with PHEV plus 50 μg/ml Tf-AF594 on ice for 30 min, after which they were shifted to 37°C for 60 min and then fixed for uptake assay. Neuro-2a cells pretreated with 50 μM CPZ exhibited a significant decrease in Tf uptake, which occurs by CME suppression, and exhibited a 76% reduction in PHEV infection (Fig. 4A). During this period, we observed that PHEVs mostly appeared to be colocalized and transported together with Tf in the large endosomal structures. Through an internalization assay, we found that the entry efficiency of PHEV was significantly decreased, to 10.6%, at 50 μM, the highest working concentration of CPZ (Fig. 4B). Furthermore, the drug stimulation significantly suppressed viral genome replication and protein translation levels as determined by qRT-PCR and immunoblotting (Fig. 4C and D). The role of CME was further tested by the expression of GFP-tagged dominant negative Eps15 mutants (Eps15EΔ95/295). Eps15 is a crucial component of CCPs, in which it interacts with adaptor protein 2 (AP-2), a major clathrin adaptor complex (33). When Neuro-2a cells overexpressing Eps15EΔ95/295 were infected with PHEV, there was a significant blockage of infection compared to that with cells expressing wild-type Eps15 (Eps15wt) or EGFP only as controls (Fig. 3D).

Clathrin is a protein that performs critical roles in the formation of coated vesicles in the cytoplasm for intracellular trafficking. It forms a triskelion shape composed of clathrin heavy chain (CHC) and light chain (CLC), and the former is known as a key component for regulating the formation and disassembly of the clathrin lattice. Here we observed that PHEVs were mostly coexpressed with clathrin at 1 h postinfection (hpi), which could indicate the presence of CCPs during CME (Fig. 4E). Since our preliminary results showed that CME is involved in PHEV entry, cell perturbation assays using small interfering RNA (siRNA)-mediated knockdown of CHC (siCHC) were carried out to evaluate the necessity of clathrin for viral infection. Twenty-four hours after transfection, PHEV entry assays were performed on transfected Neuro-2a cells, and we found that siCHC specifically decreased the expression of CHC, whereas viral internalization and proliferation were reduced in a dose-dependent manner (Fig. 4F). Meanwhile, the staining assay showed that the fluorescence signals of CHC and PHEV in 50 nM siCHC-transfected cells were reduced by approximately 84.6% and 74.5%, respectively, compared to those for the control siRNA (siCtrl)-transfected group (Fig. 4G). Taken together, these coincident results strongly suggest that PHEV entry into Neuro-2a cells can occur efficiently via CME.

### PHEV entry depends upon the fluidity of cholesterol.

**FIG 5:**
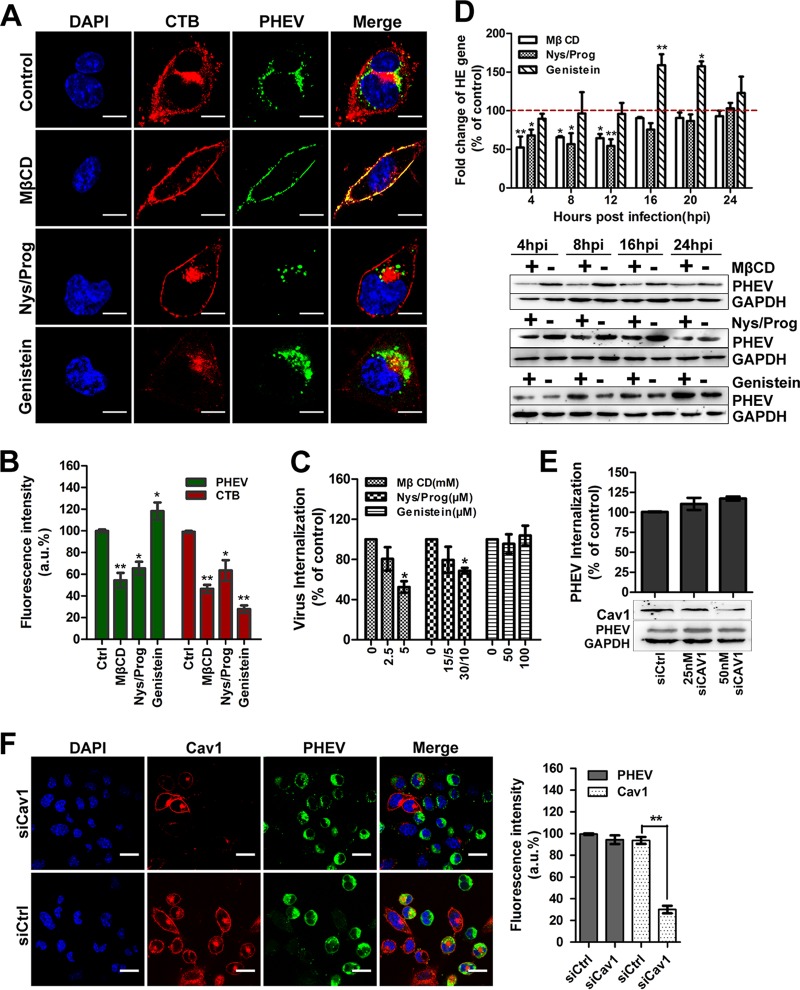
PHEV propagation depends upon cholesterol fluidity but does not involve caveola/raft-dependent endocytosis. (A) PHEV entry was assessed in cells treated with DMSO (control), MβCD (5 mM), Nys/Prog (30/10 μM), or genistein (100 μM) for 60 min. Following binding on ice, PHEV was allowed to internalize at 37°C for 60 min. Surface-bound virus was removed, and the cells were fixed and visualized by use of a confocal microscope. (B) Quantitative results for the average PHEV and CTB fluorescence intensities in cells pretreated with pharmacological inhibitors are presented in a histogram. (C) PHEV internalization after treatment of Neuro-2a cells with the indicated agents for 1 h was quantified by qRT-PCR. Data are shown as percentages of PHEV uptake compared to that of control-treated cells. (D) PHEV infection assays were carried out with MβCD-, Nys/Prog-, and genistein-treated cells. PHEV genome equivalents were estimated by qRT-PCR, using the 2^−ΔΔ*CT*^ method, while viral protein synthesis levels were estimated by Western blotting with anti-PHEV-S antibody. GAPDH was used as a loading control. (E) A PHEV uptake and propagation assay was carried out with siCav1-transfected Neuro-2a cells. Infected cells were lysed to quantitate viral RNA copy numbers by qRT-PCR, and the silencing efficiency of siCav1 was analyzed by Western blotting using anti-caveolin-1 antibody. (F) The lack of involvement of the caveola/lipid raft-mediated route was demonstrated by the stability of PHEV infection in siCav1-transfected cells. Pretransfected cells were incubated with PHEV for 24 h to allow virus propagation. The cells were then fixed, stained with anti-Cav1 (red) and anti-PHEV (green) antibodies, and visualized by confocal microscopy. Data shown are means ± SD for three independent experiments. *, *P* < 0.05; **, *P* < 0.01. Bars, 10 μm.

Most endocytic processes are sensitive to cholesterol perturbation, with both CME and caveola/lipid raft endocytosis being inhibited by removal of cholesterol (34, 35). Cholera toxin subunit B (CTB) binds to the GM1 ganglioside in lipid rafts and travels via the caveosome for delivery to the Golgi apparatus; consequently, GM1 is used as a tracer marker for caveola/lipid raft endocytosis (36). Cells were treated with a dimethyl sulfoxide (DMSO) control, methyl-β-cyclodextrin (MβCD), nystatin-progesterone (Nys/Prog), or genistein for 60 min before PHEV plus CTB incubation on ice; PHEV was allowed to internalize at 37°C for 60 min and then subjected to microscope assay. Distinct endocytic compartment structures were labeled for PHEV (green) and CTB (red) in our study, showing the sorting of these two ligands for delivery to distinct endosomal populations, which suggests that PHEV and CTB take different intracellular targeting routes to the Golgi apparatus or the endoplasmic reticulum (ER) in the perinuclear region (Fig. 5A, upper panels). Caveola/lipid raft endocytosis is known to be sensitive to nonacute cholesterol depletion induced by pharmacological agents, such as MβCD or Nys/Prog. Thus, we selectively extracted membrane cholesterol from the plasma membrane by treating Neuro-2a cells with MβCD before PHEV was added. We found that 5 mM MβCD treatment resulted in a noticeable shift of CTB from a punctate cytoplasmic distribution to being located predominantly in the plasma membrane, and quantitative analyses revealed a significant reduction (53%) in the amount of internalized CTB, indicating that the cholesterol-dependent pathway was effectively blocked (Fig. 5A and B). In this depletion assay, as observed by both internalization (Fig. 5C) and immunofluorescence (Fig. 5A and B) assays, PHEV entry was suppressed by approximately 48%, though not abolished, and the majority of both PHEV and CTB was settled on the outside surface of the plasma membrane. When cholesterol-depleted Neuro-2a cells were infected with PHEV, we found that the perturbation of cholesterol fluidity not only inhibited viral genome replication but also affected viral protein synthesis (Fig. 5D). Similar to what was observed in the MβCD assay, the uptake of CTB was blocked by pretreatment with Nys/Prog, and some of it stayed outside the plasma membrane without being internalized, while PHEV entry, replication, and proliferation were notably inhibited (Fig. 5A to D). These data confirm that PHEV enters via a cholesterol-sensitive endocytic route.

Caveola/lipid raft endocytosis occurs via signaling machinery—that is, the binding of viral cargo to the cell surface activates a tyrosine kinase-based signaling cascade, resulting in slow but efficient internalization (37). Thus, we used a well-known tyrosine kinase inhibitor, genistein, to disrupt the complex pathway and checked for the uptake of PHEV and CTB. We observed that cells pretreated with 100 μM genistein showed a significant reduction (73.6%) in CTB internalization but a slight facilitation of PHEV infection (Fig. 5A to D). This observation implies that the signaling cascade in the caveola/lipid raft-dependent pathway is a negative regulator of PHEV aggregation and that PHEV entry probably occurs via a caveola/lipid raft-independent route. To further verify this speculation, we examined the role of caveolae during viral infection. Given that caveolin-1 (Cav1) is essential for the formation and stability of caveolae and represents a scaffolding molecule for several signaling molecules, a small interfering RNA targeting caveolin-1 (siCav1) was used in this study to characterize caveolar invaginations. The efficiency of the knockdown was first validated by Western blotting (Fig. 5E), followed by a PHEV entry assay. As expected, reduction of Cav1 expression by RNA interference (RNAi) did not significantly affect viral internalization and infection in the transfected cells, suggesting that PHEV did not hijack the caveola/lipid raft-mediated route to gain entry into Neuro-2a cells (Fig. 5E and F). Taken together, these results revealed that PHEV entry is dependent on the fluidity of cholesterol but does not involve caveola/lipid raft-dependent endocytosis.

### PHEV binding on cells leads to active actin rearrangement.

**FIG 6:**
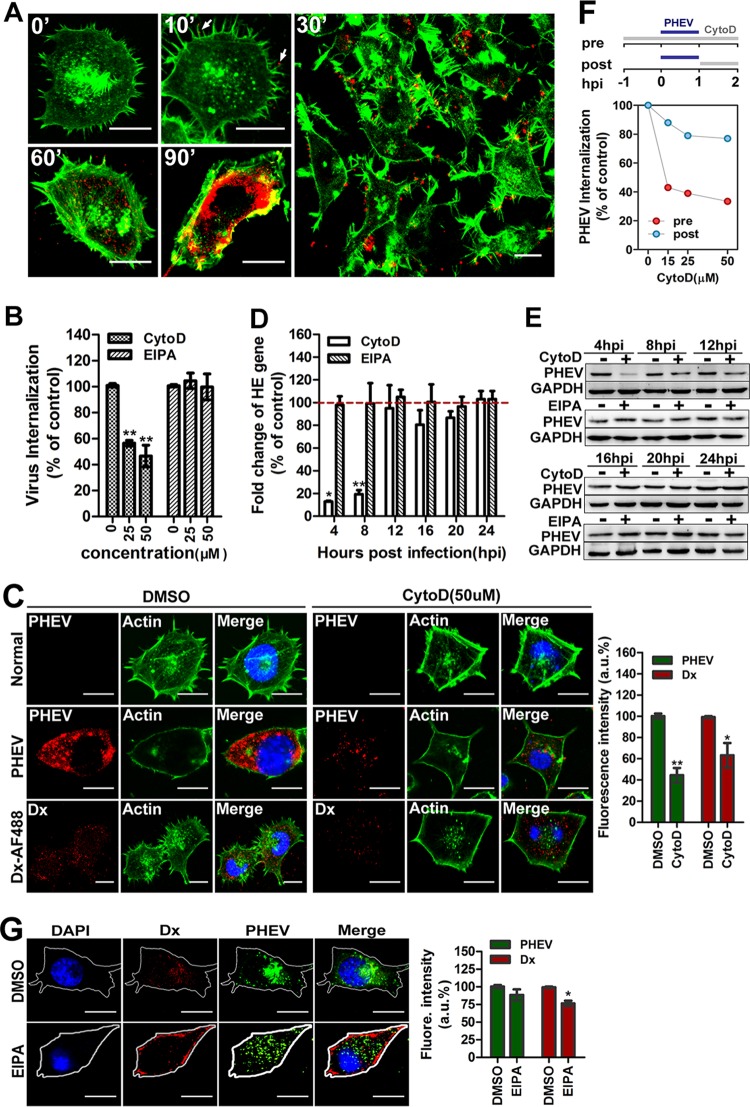
PHEV entry into Neuro-2a cells is dependent on the cytoskeleton. (A) Detection of colocalization between PHEV and actin. Neuro-2a cells were infected with DiD-labeled PHEV (red) at 37°C for the indicated times, fixed, and stained for actin by use of Alexa Fluor 488-phalloidin (1:500; green) for 30 min. At 10 min postinfection, multiple virus particles (red) bound to actin-rich protrusions, and 20 min later, PHEV particles were colocalized with actin filaments near the cell membrane during the early stage of PHEV infection. PHEV particles were found to localize in dot-like actin filaments at approximately 90 min postinfection, and images were captured immediately. (B) Neuro-2a cells were left untreated or pretreated with CytoD or EIPA at the indicated concentrations for 1 h and then subjected to PHEV internalization assays. The entry efficiency was examined by qRT-PCR, and the data shown are means ± SD for three independent experiments. (C) Neuro-2a cells were incubated under control conditions or pretreated with CytoD at 50 μM for 1 h and then subjected to a PHEV infection assay. In the uptake assays, Dx-AF488 was added for 30 min on ice, after which the cells were fixed and processed for antibody staining. Representative micrographs and quantitative analyses of average fluorescence intensity showed that PHEV infection and Dx-AF488 uptake were inhibited by CytoD and that the cells took on a more rounded shape with a smooth margin, while transient blebs filled with actin formed at the cell surface. (D) The number of PHEV genome equivalents was estimated by qRT-PCR after CytoD or EIPA treatment of Neuro-2a cells. (E) After suppression of PHEV protein synthesis by pretreatment with CytoD or EIPA for 1 h, cell lysates were collected and analyzed for the expression level of the major viral S protein by Western blotting. (F) Cells were treated with various concentrations of CytoD to inhibit polymerization of actin. As shown in the schematic, the drug was added either 60 min prior to a 1-h inoculation with virus or at 1 hpi and maintained until analysis at 4 hpi. (G) Neuro-2a cells were left untreated or pretreated with EIPA at 50 μM for 1 h and then subjected to PHEV infection and Dx-AF488 uptake assays. Images from a representative experiment out of three independent replications showed that EIPA marginally reduced Dx-AF488 uptake but did not obviously affect PHEV infection. Bars, 10 μm (A, C, and G). *, *P* < 0.05; **, *P* < 0.01.

Apart from the clathrin- and caveola/raft-dependent endocytic processes, macropinocytosis is a unique mode of endocytosis that has received increasing attention because of its roles in immune defense and virus entry. Recent studies indicated that the actin cytoskeleton is involved in the regulation of multiple endocytic pathways; in particular, F-actin polymerization is critical for the formation of outward protrusions of the plasma membrane during macropinocytosis (38). To test whether PHEV interacts with protrusions and undergoes rapid actin-driven transport to entry sites in the cell body, Neuro-2a cells were incubated with PHEV labeled with the lipophilic fluorescent dye DiD (DiD-PHEV) and fixed at various time points, and then actin was examined using phalloidin staining. Representative micrographs are shown in Fig. 6A. Without PHEV infection (0 min), the Neuro-2a cells produced random filopodial protrusions and showed a smooth margin at cell surfaces with peripheral actin staining. During the first 10 min postinfection, we found that DiD-PHEV was associated with the protrusions and the bases of the filopodia at the cell surface but that its density decreased at later time points. At approximately 30 min postinfection, the membrane-annulus phase began, and the bound viruses “surfed” toward the foot of the filopodia, located on the cell body, through actin retrograde flow. At this time, actin cytoskeleton depolymerization occurred, with the number of actin stress fibers decreasing, the cells becoming more rounded, and transient blebs filled with actin forming at the cell surface. Within 60 to 90 min, the annulus disappeared and actin accumulated in blebs and flaky pseudopods, while most of the DiD-PHEV was transported to the perinuclear cisternae. These data showed an increased distribution of actin cytoskeleton flow in cells after PHEV uptake, implying that viral entry is likely to be an actin- and molecular motor-driven process.

We further examined the involvement of actin in PHEV entry by using cytochalasin D (CytoD), which reversibly induces depolymerization of existing filaments and increases the cellular pool of ADP-bound actin monomers (39). Neuro-2a cells were pretreated with CytoD or 5-(*N*-ethyl-*N*-isopropyl) amiloride (EIPA) at the indicated concentrations for 1 h, and then PHEV internalization was examined by qRT-PCR. We observed that PHEV internalization was blocked when actin polymerization was inhibited in Neuro-2a cells (Fig. 6B). A confocal microscopy assay highlighted that pretreatment with CytoD strongly disrupted actin polymers, inhibited PHEV infection in Neuro-2a cells, and blocked the uptake of Dx-AF488, a soluble fluorescent tracer of actin-dependent pathways (Fig. 6C). As a control group, Neuro-2a cells were pretreated with DMSO and processed for later study. Meanwhile, PHEV RNA replication and protein synthesis were also reduced by the drug at the early stage, i.e., before 12 hpi (Fig. 6D and E), suggesting that functional actin cytoskeleton reorganization is required for this process. We therefore examined which stage of internalization was being affected by actin depolymerization. As shown in the schematic in Fig. 6F, CytoD was added either 60 min prior to a 1-h inoculation with virus or at 1 hpi and maintained until analysis. PHEV internalization was assayed by qRT-PCR at 2 hpi. Inhibition of actin polymerization prior to the addition of virus resulted in a 67% reduction in PHEV infection. Treatment of cells with CytoD at 1 h postinoculation had a negligible effect on PHEV accumulation as detected, confirming that the requirement for actin is specific to the very early stage of virus internalization, that is, before 1 h postinoculation (Fig. 6F).

The minimal set of requirements to satisfy the definition of virus macropinocytosis have been outlined and include actin dynamics, Na^+^/H^+^ exchanger (NHE) activity, and others. As the participation of NHE is critical for the formation of macropinocytic protrusions, we used the inhibitor EIPA, which specifically inhibits the exchange of intracellular H^+^ with external Na^+^, to clarify the role of macropinocytosis in PHEV entry. Treatment with 50 μM EIPA significantly reduced the uptake of Dx-AF488 in the cytoplasm while showing a marginal effect on internalization, but no difference in PHEV entry or infection was observed in the EIPA-treated cells by qRT-PCR, Western blotting, or IFA (Fig. 6B, D, E, and G). These data provide strong evidence suggesting that the entry of PHEV into Neuro-2a cells relies critically on actin reorganization but is independent of macropinocytosis.

### Intracellular trafficking and the role of Rabs in PHEV internalization.

**FIG 7:**
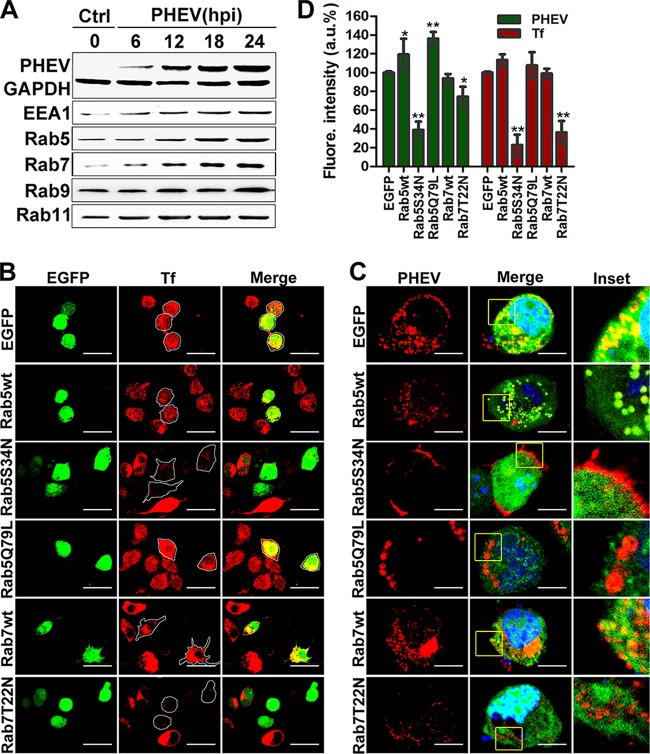
Rab5 and Rab7 are necessary for PHEV infection. (A) Neuro-2a cells were infected with PHEV, and the lysates were harvested after incubation for the indicated times and then subjected to Western blotting to detect the distribution of intracellular markers and the presence of PHEV particles. (B) Neuro-2a cells were transfected with plasmids expressing EGFP-tagged Rab5 and Rab7 WT, AN, and DN constructs and then incubated with Tf-AF488 for 30 min at 37°C. The uptake of Tf-AF488 was determined by confocal microscopy, and representative micrographs are shown. Bars, 50 μm. (C) Cells pretransfected with the indicated plasmids, pictured as in panel B, were then screened for PHEV infection. At 24 hpi, the cells were fixed, probed with anti-PHEV-S antibody, and visualized by confocal microscopy. Bars, 10 μm. (D) The effects of Rab5 and Rab7 WT, AN, and DN constructs on Tf uptake and PHEV infection were analyzed quantitatively using ImageJ software, and the arithmetic means of data from three independent experiments are shown. *, *P* < 0.05; **, *P* < 0.01.

The Rab family of small GTPases is known to play crucial roles in orchestrating membrane traffic and localizing cargos to specific subcellular compartments (19). Generally, after detaching from the plasma membrane, most enveloped RNA virus particles trafficked into EEs, marked by Rab5 and EEA1, and were then either sorted into recycling endosomes (REs), marked by Rab11, or directed to late endosomes (LEs) and endolysosomes (Lys), which are Rab7 and Rab9 positive, respectively. Therefore, we first analyzed the expression of these corresponding marker molecules by Western blotting using specific antibodies, and we found that the expression of Rab5, EEA1, Rab7, and Rab9 was significantly enhanced in the presence of PHEV, while Rab11 expression was not significantly affected (Fig. 7A). To determine whether PHEV particles were tightly associated with endosomal trafficking, we labeled EEs and LEs by transfection with EGFP-Rab5wt and EGFP-Rab7wt constructs, and their functionality was confirmed in Neuro-2a cells, with labeled transferrin (Tf-AF488) serving as a control (Fig. 7B). Next, we employed transfected cells to study PHEV entry, and we found that virus particles were colocalized with Rab5 and Rab7 during infection, indicating that Rab5 and Rab7 are involved in PHEV infection (Fig. 7C).

**FIG 8:**
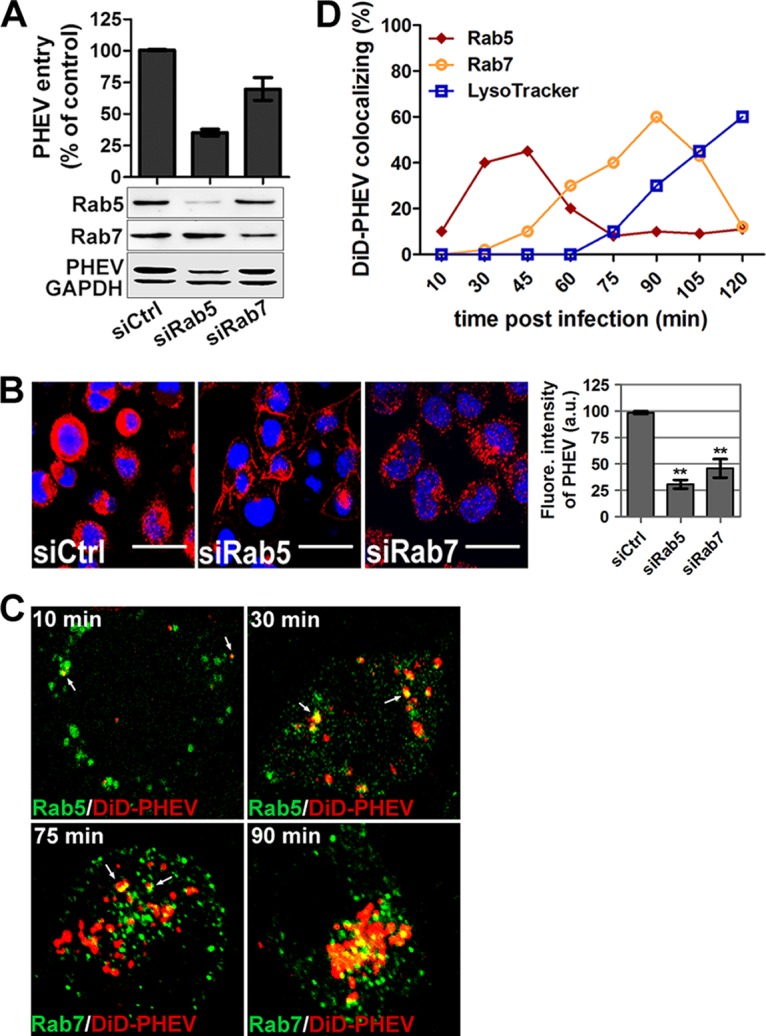
Rab5 and Rab7 are involved in PHEV intracellular trafficking. (A) Neuro-2a cells pretransfected with 50 nM siCtrl, 25 nM siRab5, or 50 nM siRab7 for 48 h were infected with PHEV and incubated for 24 h to allow virus propagation. The knockdown efficiency and viral expression were determined by Western blotting using anti-Rab5, anti-Rab7, or anti-PHEV antibody. Additionally, the infected cells were lysed to quantitate viral RNA copy numbers by qRT-PCR, and the results are presented as means ± SD for data from three independent experiments. (B) Cells transfected with the indicated siRNAs were infected with PHEV. The cells were fixed, stained with anti-PHEV antibody and DAPI, and then visualized by confocal microscopy. Bars, 20 μm. (C) Neuro-2a cells pretransfected with different EGFP-tagged endosomal markers or preprobed with LysoTracker were then exposed to DiD-labeled PHEV (red) on ice for 10 min. After being shifted to 37°C for the indicated times, the cells were washed twice with HS buffer and imaged alive at the indicated time points. Representative micrographs show that DiD-PHEV moved together with Rab5- or Rab7-positive endosomes before 45 min postinternalization, and colocalization with endolysosomes was widely seen by 90 min postinternalization, indicating that PHEV infection requires trafficking through EEs and LEs and that the virus indeed reached the endolysosomes. (D) Percentages of DiD-PHEV (red) colocalized with the endosomal markers EGFP-Rab5wt and EGFP-Rab7wt at different times after warming. The means ± SD of data from each time point for 5 to 10 cells from three different experiments are shown.

To further confirm this observation, we transfected Neuro-2a cells with the GTP-binding-defective DN mutants EGFP-Rab5S34N and EGFP-Rab7T22N and found that the uptake of the Tf-AF488 tracer was largely reduced when these mutants were overexpressed (Fig. 7B). Transfection with EGFP-Rab5S34N obviously blocked the trafficking of PHEV cargos into the cytoplasm, leaving them just beneath the cytoplasmic membranes of the infected cells, while only a moderate decrease was observed in EGFP-Rab7T22N-overexpressing cells, indicating that Rab5 and Rab7 were required (Fig. 7C). In the above-mentioned overexpression studies, the average fluorescence signals of PHEV and Tf-AF488 were quantified, and the resulting values are summarized in a histogram (Fig. 7D). We then identified the endosomal compartments traversed by endocytosed PHEV when Rab5 and Rab7 were knocked down by RNAi as a means to investigate the necessity of EEs and LEs for viral entry. Neuro-2a cells were pretransfected with 50 nM siCtrl, 25 nM siRab5, or 50 nM siRab7 for 48 h, followed by PHEV infection, and cell lysates were harvested at 24 hpi. Western blotting confirmed that Rab5 and Rab7 levels in Neuro-2a cells were efficiently knocked down by the siRNAs, and the synthesis of PHEV proteins was inhibited compared to that in siCtrl-transfected cells (Fig. 8A). These results were confirmed by qRT-PCR and IFA. We observed a significant reduction in viral entry and infectivity in siRab-transfected cells (Fig. 8A and B), which suggests the involvement of Rab5- and Rab7-mediated transport in PHEV internalization and productive infection.

To investigate the endocytic trail followed by PHEV, we allowed fluorescently labeled DiD-PHEV particles to bind to Neuro-2a cells pretransfected with the Rab constructs. As shown in Fig. 8C and D, the viral signal was captured in the cytoplasm, and DiD-PHEV-containing organelles had acquired or fused with Rab5-positive EEs at approximately 30 min postinternalization. Seventy-five minutes later, the cotransport of Rab5 and PHEV gradually disappeared, while a few DiD-PHEV particles were detected in Rab7-positive LEs. By 90 min postinternalization, this colocalization of Rab7 and PHEV was widely apparent. Given this cotransport of DiD-PHEV particles with Rab5 and Rab7 in the cytoplasm, we concluded that Rab5-positive EEs and Rab7-positive LEs were required for viral transport, undergoing endosomal maturation to initiate an infectious cycle.

### PHEV infection is dependent on Rab5 activation.

The Rab family of GTPases, whose activity depends on GDP/GTP association, plays a central role in intracellular transport. In general terms, GTP binding to Rabs allows the recruitment of effectors that mediate vesicular movement on the cytoskeleton and fusion with membranes, and the processes are reversible and bidirectional. Earlier work showed that Rab5 depletion has a larger effect than that of Rab7 depletion on PHEV entry and transport (see above), suggesting that the functionality of Rab5 may play a more important role in PHEV infection. Thus, we directly examined the possibility that PHEV may facilitate Rab5 activity during internalization and intracellular trafficking.

**FIG 9:**
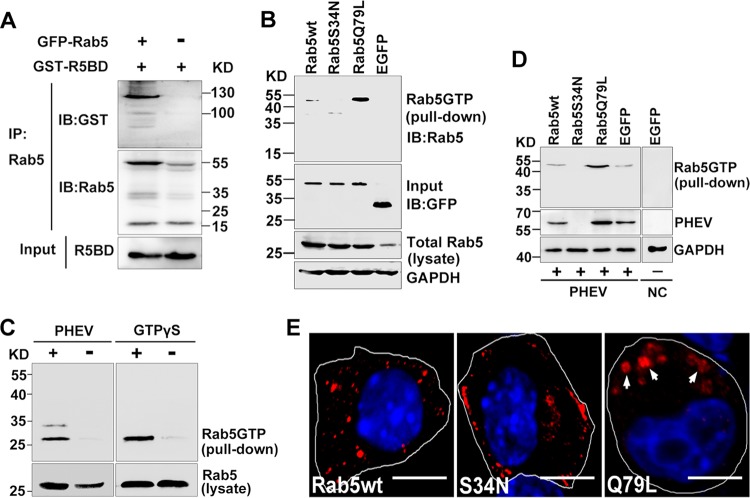
Endocytosis of PHEV is dependent on Rab5 activation. (A) Coimmunoprecipitation of Rab5 with Rabaptin5. Neuro-2a cell lysates containing coexpressed GFP-Rab5 and GST-R5BD were immunoprecipitated (IP) with anti-Rab5 antibody-conjugated Sepharose beads, followed by immunoblot (IB) analysis with anti-Rab5 and anti-GST antibodies. Control cells expressed Rabaptin5 only in the absence of Rab5. (B) Neuro-2a cells were transfected with plasmids expressing Rab5wt, Rab5S34N, Rab5Q79L, and EGFP-N, as indicated, after which they were subjected to cell lysis and pulldown assays as described below for panel C. Immunoblots of GAPDH and GFP on the same membrane serve as the loading control and input control, respectively. (C) Rab5 activation assay. Neuro-2a cell lysates that were either left untreated or pretreated with PHEV were then incubated with GST-R5BD. The GTP-bound Rab5 fraction bound to GST-R5BD (pulldown) and the total Rab5 protein in the lysates were purified by SDS-PAGE and subjected to IB analysis with anti-Rab5 antibody (left panels). As a control, purified full-length Rab5 proteins were immunoprecipitated after being treated with 0.2 mg/ml GTPγS at 37°C for 45 min (right panels). (D) Cells expressing Rab5wt, Rab5S34N, Rab5Q79L, and EGFP-N were used for a PHEV propagation assay. At 12 hpi, the cells were lysed and harvested for immunoblot analysis with the indicated antibodies. (E) Cells expressing the indicated constructs were treated with Tf-AF488 for 30 min at 37°C. Endocytosis of Tf-AF488 was determined by confocal microscopy, and representative micrographs are shown. Bars, 10 μm.

Given that Rab5 is a GTPase, its activity in cells was monitored by the level of active GTP-bound Rab5 (Rab5-GTP), which we determined using a glutathione *S*-transferase (GST) pulldown assay. Because of the specific binding of Rab5-GTP by the C-terminal Rab5-binding domain (R5BD) of the effector Rabaptin5 (40), a GST-R5BD fusion protein was generated to pull down Rab5-GTP in this study. Prior to that step, the interaction of GST-R5BD was determined by coimmunoprecipitation (Fig. 9A). As positive and negative controls, the constitutively active mutant Rab5Q79L and the GTP-binding-defective mutant Rab5S34N, respectively, were transfected into cells, followed by GTPase activity detection using a GST-R5BD pulldown assay. Overexpression of Rab5Q79L produced the most robust pulldown signal, which reflected high GTPase activity, while the signal was completely inhibited after transfection with the DN isoform Rab5S34N, demonstrating the feasibility of the GST-R5BD pulldown assay for determining the relative amount of Rab5-GTP in the cell (Fig. 9B). Based on this, we then proceeded to perform a Rab5 activation assay to determine the relative amount of Rab5-GTP underlying PHEV infection. The endogenous Rab5-GTP level was too low to be detected. Therefore, a nonhydrolyzable GTP analog, guanosine gamma thiophosphate (GTPγS), was added to activate small G proteins as a positive control. We found that PHEV rapidly upregulated activation of endogenous Rab5 at 1 h postinfection (Fig. 9C), indicating that Rab5-GTP played a role in this process.

To determine whether PHEV invasion may be affected by the Rab5-GTP level, we overexpressed the Rab5 wild-type (Rab5wt), dominant active (DA; Rab5Q79L), and dominant negative (DN; Rab5S34N) constructs in Neuro-2a cells, followed by PHEV infection. At 12 hpi, the cells were lysed and harvested for immunoblot analysis with the indicated antibodies. As expected, overexpression of the high-GTPase-activity isoform Rab5Q79L facilitated PHEV RNA replication and proliferation, while overexpression of the DN isoform Rab5S34N significantly inhibited productive infection (Fig. 9D). In the Rab5wt-transfected group, cells showed an increased pulldown signal, but to a much lesser extent than that with Rab5Q79L (Fig. 9C), whereas PHEV infection was 7-fold higher than that in the EGFP-transfected group (Fig. 9D). Profiles similar to the above findings were observed by IFA: Rab5Q79L overexpression not only increased PHEV infection but also formed some sorting-defective and enlarged Rab5-positive vesicles that allowed us to more easily detect virion particles, reinforcing the notion that the virus traffics through Rab5-positive EEs (Fig. 7C). These results were further evidenced by increased endocytosis of Tf-AF488: overexpression of Rab5Q79L induced the formation of enlarged vesicles that recruited excessive accumulation of Tf-AF488 compared to that in cells expressing Rab5wt (Fig. 9E). In contrast, expression of Rab5S34N reduced Tf-AF488 uptake to 66% of control levels (Fig. 9E). Together, these results suggest that the GTPase Rab5 is critical for PHEV propagation and that endogenous Rab5 function may need to be activated during viral entry and intracellular trafficking.

## DISCUSSION

Porcine hemagglutinating encephalomyelitis is an acute and highly contagious disease that is caused by PHEV and leads to vomiting, depression, pallor, and dehydration as well as neurologic signs, such as abnormal gait, dullness, anorexia, tremors, and nystagmus. Since 1958, when the disease first broke out (41), PHEV infection has been reported successively in several pig-raising countries, including the United States, Japan, Argentina, Belgium, South Korea, and China (2–4, 42–44). Though the epidemiology and pathogenesis of PHEV were gradually characterized, little information is available about its infectious cell entry pathway. This study is the first investigation of the cell entry process of PHEV particles. Moreover, we revealed that Rab5 and Rab7 are involved in PHEV invasion and intracellular trafficking in Neuro-2a cells.

Understanding the viral entry pathway, a very early and critical step in the infection of host cells, is important for the understanding of viral pathogenesis and design of antiviral drugs (45). Coronavirus entry is largely controlled by the S glycoprotein, since it bears both receptor binding and membrane fusion capabilities. During the initial receptor binding, large conformational changes of the S protein have evolved. Depending on the coronavirus species and strain, the S protein can mediate binding to a proteinaceous receptor or to carbohydrate moieties, and thereby, a multitude of mechanisms and strategies of virus entry have been exploited. For example, MHV strains are capable of entering directly at the cell surface or through the endocytic pathway (46, 47). While SARS-CoV was initially thought to enter cells through direct fusion with the plasma membrane, more recent evidence suggests that virus entry may also occur through a novel clathrin- and caveola-independent endocytic pathway (48). Increasing evidence has demonstrated that the choice of coronavirus entry mechanism is very complex. As such, this study on PHEV entry mechanisms is notable and increasingly central for understanding the infection process.

Of the endocytic pathways taken by viruses, the most commonly used is the CME route. Clathrin is first recruited to assemble CCPs on the cytoplasmic side of the membrane in response to receptor-mediated endocytosis signals, and it is composed of a light chain (CLC) and a heavy chain (CHC) (49). In our study, RNA interference assays indicated that clathrins are required for the PHEV-induced formation of CCPs at their binding sites. Once assembly is finished, CCPs pinch off from the plasma membrane to form clathrin-coated vesicles (CCVs), which involves the dynamic function of dynamin-2 around the neck of the endocytic indentations. As for large GTPases, dynamin-2 is required for various types of endocytosis and intracellular scission events and is indispensable for CME (50). We found that PHEV endocytosis has a dynamin-2- and low-pH-dependent nature, prompting us to hypothesize that PHEV entry is a quick process, in accordance with the characteristics of CME. As expected, disrupting the assembly of clathrin lattices and blocking AP-2 recruitment to CCPs decreased PHEV entry, showing that it occurs via CME.

Cholesterol is essential for the structure of invaginated caveolae and CCPs within the cell membrane and thereby functions in both caveola- and clathrin-dependent endocytosis (35). Caveola/lipid raft-mediated endocytosis is characterized by caveolae, which are smooth, cholesterol- and sphingolipid-rich invaginations of the plasma membrane and are generally associated with caveolin (21). Our data showed that cholesterol depletion suppressed PHEV infection, implying that the fluidity of cholesterol is important during PHEV entry and replication, though not as the most pivotal factor. Interestingly, the tyrosine kinase inhibitor used to disrupt caveola function did not affect PHEV infection. Together with the fact that knockdown of caveolin-1 was barely an obstacle to PHEV proliferation, our findings suggest that PHEV entry occurs not through disabling caveola-mediated endocytosis *per se* but most likely by altering the integrity of membrane lipid microdomains.

After binding, many viruses move laterally along the cell surface before internalization, followed by binding to filopodia and “surfing” toward the cell body through actin retrograde flow (51). For PHEV, we observed that virus particles were located in the actin-rich protrusions and then moved toward colocalization with dot-like actin and long actin filaments. Subsequently, PHEV induced rearrangements of the actin cytoskeleton, pointing to a greater role for the actin cytoskeleton in viral entry and transport. To date, actin cytoskeleton involvement in PHEV internalization has clearly been demonstrated, but data showing its specific contribution are still missing.

**FIG 10:**
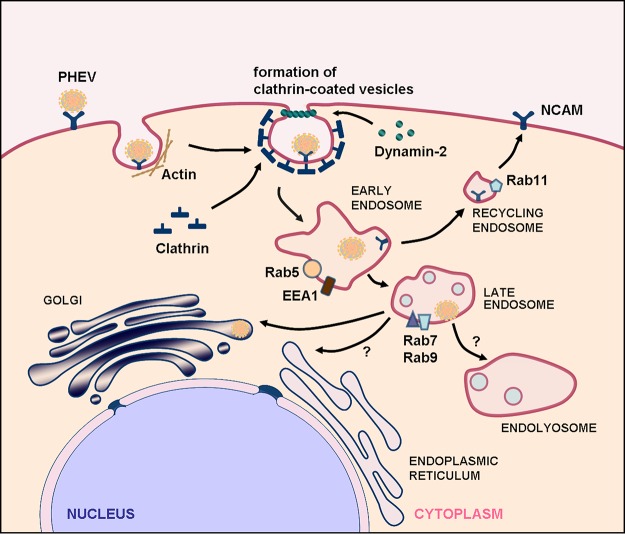
Models of cellular entry and trafficking of PHEV in Neuro-2a cells. PHEV particles may be transported along actin-rich protrusions to reach the cell surface, and internalization from the plasma membrane may be induced by the classical mechanism of CME. The individual particles are rapidly taken up, internalized into CCVs, and then transported to Rab5-, EEA1-, and NCAM-positive EEs in a clathrin-, dynamin-2-, Eps15-, and Rab5-dependent manner. Through endosome maturation, these virus-containing endosomes acquire Rab7 and become luminal components of Rab7- and Rab9-positive LEs. Further transport to the Golgi apparatus occurs from the late compartments of the endocytic pathway, by an unknown mechanism, and the recycling endosomes are not essential in this process. The vacuolar ATPase (v-ATPase) is responsible for the acidification of the endosomal system, a process that is required for PHEV internalization and the subsequent transport steps. Early and late events in the entry pathway can be blocked by various inhibitors and other perturbants.

Once internalized within the primary endocytic vesicles, many viruses are delivered to suitable endosomes or other intracellular organelles and follow the intracellular pathways of the endosomal/lysosomal system, which is responsible for molecular sorting, recycling, degradation, storage, and processing. Owing to restricted expression patterns in specific endocytic compartments and the ability to recruit distinctive effectors, Rab GTPases are powerful tools for discriminating between pathways leading to different intracellular locations. In the present study, we monitored the intracellular trafficking following endocytosis and showed that PHEV infection is regulated by EEs, LEs, and endolysosomes, while the involvement of recycling endosomes in this process is uncertain (Fig. 10). Rab activity plays roles in endocytosis (52). In general, Rab cycles between GTP-bound and GDP-bound states, and the conversion between these two states is regulated by GTPase-activating protein, GDP exchange factor, GDP dissociation inhibitor (GDI), Rab escort protein (REP-1), and some effector molecules. Here we overexpressed the constitutively active mutant Rab5Q79L and the GTPase-deficient mutants Rab5S34N and Rab7T22N and clearly demonstrated that Rab5 activity and functional early endocytic pathways are required for effective PHEV infection. However, whether PHEV infection depends on lysosomal activity remains unexplored.

In summary, we conducted the first systematic study to dissect the internalization and intracellular trafficking mechanism of PHEV in Neuro-2a cells, and these findings are highly relevant for the development of future therapies directed at PHEV. To date, there are no specific antiviral drugs that have been shown to effectively treat PHEV infection. A better understanding of the molecular pathogenesis involved in porcine hemagglutinating encephalomyelitis is likely to open new approaches for the development of novel therapeutic strategies. However, for many aspects of PHEV infection, the underlying physiological processes remain to be characterized. To move forward, it is important to take advantage of powerful novel technologies, combined with improved assays for following a virus step by step through its replication cycle, to achieve unprecedented access to the underlying network of cellular factors involved.

## MATERIALS AND METHODS

### Cells, virus, reagents, and antibodies.

Neuro-2a cells were grown in Dulbecco's modified Eagle's medium (DMEM) supplemented with 10% fetal bovine serum (HyClone) and 1% penicillin-streptomycin. The PHEV strain used in all experiments was HEV 67N (GenBank accession number AY078417), which was successfully propagated in Neuro-2a cells (CCL-131; ATCC). The Neuro-2a cell line is sensitive to infection with PHEV and has been used extensively for virus infection and replication studies. For PHEV entry and replication determinations, viral infection was performed by inoculating PHEV (multiplicity of infection [MOI] = 50) into Neuro-2a cell cultures for the indicated times before RNA isolation or cell lysis. All endocytic inhibitors, i.e., chlorpromazine (CPZ), genistein, methyl-β-cyclodextrin (MβCD), nystatin (Nys), progesterone (Prog), ammonium chloride (NH_4_Cl), chloroquine (CQ), cytochalasin D (CytoD), 5-(*N*-ethyl-*N*-isopropyl) amiloride (EIPA), and dynasore, were purchased from Sigma. Antibodies against NCAM, EEA1, Rab5, Rab7, Rab9, Rab11, and caveolin-1 were purchased from Cell Signaling Technology. An antibody against Rabaptin5 was purchased from Abcam. Alexa Fluor 594-conjugated transferrin (Tf-AF594), Alexa Fluor 555-conjugated cholera toxin subunit B (CTB-AF555), and Alexa Fluor 488-conjugated dextran (Dx-AF488) were all obtained from Invitrogen. GTPγS and phalloidin were purchased from Cytoskeleton, Inc. (Denver, CO). For this study, a mouse anti-PHEV-S antibody (monoclonal antibody 1E2) which weighs about 66.4 kDa and recognizes the S protein of PHEV was used in all Western blot and immunofluorescence analyses.

### qRT-PCR and Western blotting.

Total RNA was extracted using TRIzol reagent (Invitrogen), and reverse transcription was performed using PrimeScript reverse transcriptase (TaKaRa, Japan). qRT-PCR was conducted with FS Universal SYBR green master mix (Roche) on a CFX96 Touch real-time PCR detection system (Bio-Rad, USA), with glyceraldehyde-3-phosphate dehydrogenase (GAPDH) as a control for the purpose of normalization. For Western blotting, cells were washed three times with phosphate-buffered saline (PBS) and lysed in RIPA lysis buffer (1% Triton X-100 and 1 mM phenylmethylsulfonyl fluoride [PMSF] in PBS) on ice. After being separated by SDS-PAGE, the proteins in the lysates were electrotransferred to polyvinylidene difluoride (PVDF) membranes and then immunoblotted with the indicated antibodies. GAPDH was used as a loading control.

### Assay of PHEV internalization kinetics.

To test internalization, we prechilled Neuro-2a cells for 10 min and then exposed them to PHEV on ice for 30 min for virus binding. Ice-cold PBS (0.1 M, pH 7.2) was added to completely remove unbound viruses, and then the cells were shifted to 37°C to allow internalization. After the cells were incubated for the indicated time spans, they were treated with citrate buffer (40 mM sodium citrate, 10 mM KCl, and 135 mM NaCl, pH 3.1) for 30 s to inactivate and remove noninternalized viruses. The cells were either fixed for visualization by microscopy with the anti-PHEV-S antibody or subjected to qRT-PCR analysis. For experiments involving inhibitor stimulation, cells were pretreated with the indicated inhibitor for 1 h before the addition of PHEV on ice, and the remaining steps were conducted as described above.

### Thin-section TEM.

Cells were plated in 6-well plates at a density of 2 × 10^5^ cells/well, grown for 24 h at 37°C, and precooled in an ice-water bath for 15 min. PHEV was added and incubated for an additional 30 min on ice, and then the cultures were shifted to 37°C. Zero, 30, and 60 min after the temperature shift, cells were collected, fixed with 2.5% glutaraldehyde for 10 min, and then postfixed in 1% osmium tetroxide for 2 h. After stepwise dehydration in ethanol, the specimens were embedded in Epon resin and polymerized at 60°C for 24 h. The ultrathin sections were stained in brass wire mesh with 2% uranyl acetate for 30 min and 0.4% lead citrate for 2 min and then visualized with a transmission electron microscope.

### Cytotoxicity assay and drug administration.

Cells were seeded in 96-well plates at a density of 2 × 10^5^ cells/well, grown for 24 h, and then treated with endocytosis inhibitors at the indicated concentrations for 1 h. After two washes with DMEM, 10 μl of CCK-8 solution was added to 100 μl of DMEM in each well of the 96-well plate and incubated at 37°C for 1 h, and the absorbance at 450 nm was measured with a microplate reader. The experiment was carried out in triplicate, and error bars in figures represent standard deviations (SD). The concentrations of all chemical reagents used in this study did not cause significant cytotoxic effects on cell viability (Table 1). Confluent monolayers of Neuro-2a cells grown in 12-well plates were washed twice with PBS and pretreated with the appropriate concentration of each drug for 60 min. The cultures were infected with PHEV for 1 h in the presence of the corresponding inhibitor and then washed twice with PBS and incubated for an additional 12 or 24 h at 37°C. At the indicated times, cells were processed for IFA, qRT-PCR, and Western blotting.

**TABLE 1:** Details of all drugs employed in this study

Drug	Solvent^*a*^	Storage temp (°C)	Working concn (μM)
Ammonium chloride (NH_4_Cl)	ddH_2_O	4	2,000, 5,000
Chloroquine (CQ)	ddH_2_O	4	30, 60
Chlorpromazine (CPZ)	ddH_2_O	4	25, 50
Cytochalasin D (CytoD)	DMSO	−20	50
Dynasore	DMSO	−20	10, 20
Genistein	DMSO	−20	50, 100
Methyl-β-cyclodextrin (MβCD)	ddH_2_O	4	2,500, 5,000
Nystatin (Nys)	DMSO	−20	15, 30
Progesterone (Prog)	DMSO	−20	5, 10
5-*N*-Ethyl-*N*-isoproamiloride (EIPA)	DMSO	4	50

a ddH_2_O, double-distilled water.

### RNA interference and transient transfections.

Plasmids for wild-type (WT) sequences and dominant active (DA) and dominant negative (DN) mutant sequences were constructed using conventional cloning techniques. To determine the infectivity of PHEV in cells, we pretransfected the plasmids into Neuro-2a cells by using X-tremeGENE HP DNA transfection reagent (Roche, Sweden) according to the manufacturer's instructions. For the RNA interference assay, siRNAs against the clathrin heavy chain (siCHC) (5′-UGACAAAGGUGGAUAAAUU-3′), caveolin-1 (siCav1) (5′-GCAGUUGUACCAUGCAUUA-3′), Rab5 (siRab5) (5′-CCAGGAAGCACAGUCCUAU-3′), and Rab7 (siRab7) (5′-GUACAAAGCCACAAUAGGA-3′) were designed based on the full-length mRNA sequences (accession numbers NM_028504.1, NM_007616.4, AB232593.1, and AB232599.1). An siRNA with a control sequence irrelevant to all known genes (siCtrl) (5′-UUCUCCGAACGUGUCACGU-3′) was also designed and synthesized. Neuro-2a cells were transfected with the appropriate siRNA by use of X-tremeGENE siRNA transfection reagent according to the manufacturer's instructions, and the knockdown efficiencies were quantified by Western blotting. Subsequent experiments were performed 48 h after transfection.

### Confocal microscopy and IFA.

Neuro-2a cells growing on glass coverslips were fixed in 4% paraformaldehyde for 10 min and permeabilized with 0.2% Triton X-100 for 10 min, after which they were blocked with 5% bovine serum albumin (BSA) for 1 h at room temperature. The cells were then probed with primary antibodies, i.e., anti-NCAM (1:200), anti-EEA1 (1:1,000), and anti-PHEV (1:200), for 1 h at 37°C. After three washes with PBS, the cells were incubated for 1 h at room temperature with secondary antibodies (anti-mouse IgG antibody conjugated to Alexa Fluor 488 or Alexa Fluor 594 and anti-rabbit IgG antibody conjugated to Alexa Fluor 594 [Molecular Probes]) at a 500-fold dilution, and then the cell nuclei were labeled with DAPI (4′,6-diamidino-2-phenylindole) (1:1,000; Sigma). For the DA or DN protein assay, cells were pretransfected with EGFP plasmids encoding Dyn2wt, Dyn2K44A, Eps15wt, EpsΔ95/295, Rab5wt, Rab5S34N, Rab5Q79L, Rab7wt, or Rab7T22N and then infected with PHEV and immunolabeled as described above. For the actin rearrangement analysis, monolayers were incubated with fluorescein isothiocyanate (FITC)-phalloidin (1:200) for 1 h at 37°C after being immunolabeled as described above, and then the percentages of cells with normal and disorganized actin filaments were determined. The specificity of labeling and the absence of signal crossover were confirmed by examination of single-labeled control samples. All images were acquired randomly from different fields of view on each coverslip, with more than 30 cells per field, using a laser scanning confocal fluorescence microscope (Olympus FluoView FV1000). Virus infection motility and subcellular colocalization were analyzed using ImageJ and MATLAB.

### Virus purification and DiD labeling.

PHEV was purified by sucrose density gradient centrifugation. The virus was first purified over 30%, 45%, and 60% sucrose cushions in an ultracentrifuge (CP100WX; Hitachi) at 80,000 × *g* for 4 h at 4°C, and then the virus was exchanged into PBS through cycles of concentration by centrifugation (20,000 × *g*, 2 h, 4°C) and dilution with PBS. Purified PHEV was freshly labeled with the lipophilic fluorescent dye DiD (4-chlorobenzenesulfonate salt; Life Technologies), a dialkylcarbocyanine analog that has markedly red-shifted fluorescence excitation and emission spectra and is widely used as a labeled tracer in living or fixed cells. Briefly, the PHEV stock solution was mixed with DiD for 10 min, and the free dye was removed using gel filtration columns (GE Healthcare) and HS buffer (2.5 mM HEPES, 145 mM NaCl). The Neuro-2a cells were infected with DiD-PHEV, and images were obtained and analyzed using a confocal microscope.

### Uptake assays.

Cells grown on coverslips were left untreated or pretreated with the corresponding inhibitors and then incubated with PHEV plus 50 μg/ml Tf-AF594, PHEV plus 50 μg/ml CTB-AF555, PHEV plus 50 μg/ml Dx-AF488, or PHEV alone on ice for 15 to 30 min, after which they were shifted to 37°C and incubated for 60 min. For the CTB and Dx assays, noninternalized PHEV, CTB-AF555, and Dx-AF488 were removed by washing the cells with PBS three times and adding 0.5 ml of room-temperature 0.25% trypsin; 1 min later, we removed the trypsin, washed the cells with cold complete medium twice, fixed them with cold 0.4% paraformaldehyde in PBS, and analyzed the fixed cells by IFA with the indicated antibodies. For the transferrin assay, we removed the substrate-containing medium by washing the cells with PBS three times and then incubating them for 2 min in cold stripping buffer (150 mM NaCl, 20 mM HEPES, 5 mM KCl, 1 mM CaCl_2_, 1 mM MgCl_2_[pH 5.5]); after washing the cells with cold PBS, we proceeded with IFA.

### GST pulldown assay.

The Rab5-binding domain (R5BD) of Rabaptin5 was first cloned into pGEX-4T-1 plasmids encoding the fusion protein GST-R5BD in Escherichia coli BL21, and expression was induced with 0.1 mM isopropyl-β-d-thiogalactopyranoside (IPTG). GST-R5BD was then affinity purified by use of glutathione Sepharose 4B beads (Amersham Pharmacia Biotech) and quantified by SDS-PAGE. Cells were transfected with Rab5 proteins (Rab5wt, Rab5S34N, and Rab5Q79L) at 37°C for 24 h, followed by treatment with PHEV, and then collected and lysed for 15 min in lysis buffer (25 mM HEPES [pH 7.4], 100 mM NaCl, 5 mM MgCl_2_, 1 mM EDTA, 1 mM dithiothreitol [DTT], 0.1% NP-40, 20% glycerol, and 0.1% protease inhibitor). To measure Rab5 GTPase activation in response to PHEV infection, the virus was preabsorbed into the cells at 37°C for 24 h, and then the cells were collected and lysed; guanosine gamma thiophosphate (GTPγS) was added to partial lysates to activate small G proteins as a positive control. After being clarified by centrifugation at 10,000 × *g* for 10 min at 4°C, the lysates were incubated with the purified GST-R5BD protein at 4°C overnight on a rotating mixer. The resin was subsequently rinsed with the lysis buffer and resuspended in SDS loading buffer, and Western blotting was subsequently performed.

### Image and statistical analyses.

All data are presented as means ± SD for at least three independent experiments, as indicated. All statistical analyses and calculations were conducted using two-tailed Student's *t* tests or one-way analysis of variance (ANOVA) in GraphPad Prism, version 5, software (GraphPad Software Inc., La Jolla, CA). A *P* value of <0.05 was defined as the threshold for statistical significance (single asterisks), and results were considered very significant for *P* values of <0.01 (double asterisks).
